# Protein intake and muscle mass of community-dwelling older adults: a cross-sectional study in Kigali, Rwanda

**DOI:** 10.1038/s41598-025-03291-2

**Published:** 2025-05-24

**Authors:** Theogene Habumugisha, Eric Matsiko, Inger Elisabeth Måren, Matthias Kaiser, Alida Melse-Boonstra, Karin Borgonjen-van den Berg, Jutta Dierkes, Ingunn M. S. Engebretsen

**Affiliations:** 1 Department of Global Public Health and Primary Care, Centre for International Health, University of Bergen, Årstadveien 21, Bergen, 5009 Norway; 2 Department of Human Nutrition and Dietetics, College of Medicine and Health Sciences, University of Rwanda, Kigali, Rwanda; 3 Department of Biological Sciences, University of Bergen, Bergen, Norway; 4 Centre for the Study of Sciences and Humanities, University of Bergen, Bergen, Norway; 5 Division of Human Nutrition and Health, Wageningen University and Research, Wageningen, The Netherlands; 6 Department of Clinical Medicine, Centre for Nutrition, and Mohn Nutrition Research Laboratory, University of Bergen, Bergen, Norway; 7 Department of Medical Biochemistry and Pharmacology, Haukeland University Hospital, Bergen, Norway

**Keywords:** Protein, Muscle mass, Animal-sourced foods, Sustainable diet, Aging, Sub-Saharan Africa, Medical research, Epidemiology

## Abstract

Older people are at high risk of protein deficiency, which accelerates the age-related loss of muscle mass. However, there is a paucity of data on the consumption of protein-rich animal-sourced foods and their relationship with dietary intake and muscle mass of older adults in low- and middle-income countries. Therefore, this study assessed ASF consumption and its associations with protein intake and muscle mass of older adults. A cross-sectional study was conducted among community-dwelling older adults in the Gasabo district, Rwanda. Dietary intake was estimated using two non-consecutive 24-h recalls. A structured questionnaire was used to collect data on socio-demographic characteristics. Inverse propensity score weighting was employed to assess the association between animal-source food (ASF) consumption and protein intake and muscle mass. Slightly more than half (56%, *n* = 189) of the older adults consumed ASFs. In adjusted models, ASF consumption was positively associated with protein intake (ATE: 9.6 g/d; CI, 6.8 to 12.4) and muscle mass (ATE: 1.7 kg; CI, 0.0 to 3.4). The results also showed that adding one and two or more ASFs to the diet was proportionally associated with an increase in protein intake (ATE: 4.7 g/d; 95% CI, 0.2 to 9.3 and ATE: 13.9 g/d; 95% CI, 5.9 to 22.0, respectively), but not with muscle mass (ATE: 1.2 kg; 95% CI − 1.4 to 3.9 and ATE: 1.4 kg; 95% CI − 0.6 to 9.6, respectively). The consumption of ASFs was generally low among community-dwelling older adults. However, older adults who consumed ASFs had improved protein intake and muscle mass compared with those who consumed plant-based diets. Large and prospective studies, extending beyond urban settings, are needed to identify factors that can be targeted to improve ASF consumption and overall dietary intake among older adults in Rwanda.

**Supplementary Information:**

The online version contains supplementary material available at 10.1038/s41598-025-03291-2.

## Introduction

A rapidly growing proportion of older adults in the population is becoming a challenge for public health in both low- and high-income settings^1^. The WHO estimates that, globally, the number of older persons (aged over 60 years) will exceed 2 billion by 2050^2^, and the majority of this population is expected to live in low- and middle-income countries (LMICs)^2^. The rise in the number of older persons is mainly due to global longevity, resulting from improvements in healthcare and living standards^1^ Globally, the average life expectancy (LE) rose from 47 years in 1950 to 72 years in 2019, and it is expected to continue rising in the coming decades, reaching 78 years in 2050^3^. Sub-Saharan Africa (SSA) has the lowest average life expectancy, which stood at 59 years in 2021^3^. Like other regions, life expectancy is expected to rise in SSA in the next few decades, reaching around 67 years in 2050^3^. In Rwanda, the life expectancy at birth has also increased, rising from 65 years in 2012 to 70 years in 2022^4^.

This forecasted demographic shift will have implications for the individual health and care of the older population. Older people often face multiple physiological and psychosocial changes that increase the risk of nutrient deficiency and malnutrition^5^. Physiological changes are related to alterations in body composition, where older people lose skeletal muscles with a concurrent increase in body fat^5^. Although the loss of muscle mass can occur at any time throughout the lifespan due to diseases like cancer^6^, the age-related decline in muscle mass has been shown to start as early as the 4th or 5th decade of life^7,8^.

Skeletal muscle is one of the most abundant and highly metabolically active tissues of the body^9^, and its loss has a major impact on the metabolism and energy requirement of older persons^10^. Moreover, the capacity of the body to synthesize muscle proteins declines with age, resulting in a higher requirement for protein intake to maintain lean body mass in older persons^11^.

Adequate intake of high-quality protein and energy is essential to sustain adequate supplies of protein for muscle synthesis^12^. However, older adults generally eat less food than young adults^13^, making it more difficult to cover their protein and calorie (energy) needs. It is estimated that older adults consume about 30% less energy per day than young adults^14^. The decline in food intake is related to physical and physiological changes with advancing age^13^. Loss of taste and smell is one of the such changes that reduce food intake by affecting appetite^13^. Aging is also associated with a reduced sense of satiety^15^, affecting the amount of food consumed by older adults.

The consumption of protein-rich foods, such as meat, dairy, fish, and eggs, provides energy and high-quality proteins to the body while consuming a low bulk of foods can help individuals overcome older age-related challenges^16^. However, for some older adults, the ability to consume adequate proteins is hindered by a reliance on foods that are inherently deficient in high-quality nutrients^17^. Plant-based foods generally contain a lower protein, fat, and energy density than animal-sourced foods (ASFs)^18^. Digestibility and bio-accessibility are also higher for animal proteins than proteins from plant-based foods^18^. Therefore, it is believed that people who consume plant-based foods may need to eat large amounts of food to obtain sufficient high-quality proteins^16^.

The implication of choosing dietary sources of protein (animal vs. plant) on health and environmental sustainability also compounds the puzzle of meeting the protein needs of the aging population^19^. A high consumption of animal proteins has been associated with a high intake of unhealthy fats and low fiber^20^, which increases the risk of cardiovascular diseases.

On the other hand, ASF consumption, particularly meat and dairy, is also responsible for a large share of food-related greenhouse gas emissions and biodiversity loss^21^. Although per capita food-related greenhouse gas (GHG) emissions are very high in HICs, recent estimates show that LMICs account for the largest cumulative emissions globally^22^. Similarly, over the past three decades, low-income countries recorded the highest increase in emissions in the agri-food system (53%), compared with middle-income countries (12.3%) and HICs (3%)^22^. Thus, high consumption of ASFs is being discouraged to limit the intake of unhealthy nutrients and protect planetary health^23^. However, it is debated whether plant-based diets should be promoted for vulnerable populations with a higher risk of deficiencies and malnutrition, such as older adults, children, and pregnant women^24,25^.

The scientific discourse, mainly from high-income countries (HICs), on replacing ASFs from the diet and shifting towards plant-based diets is centered on whether such diets can provide sufficient nutrients and preserve muscle mass in the aging population^19,26^. In these countries, there is also a diverging view on whether plant-based diets can be recommended for older populations^27^. Those who support plant-based diets argue that plant-based foods can satisfy the nutrient needs for people in all life-stages, including older persons^28,29^. High levels of protein intake in HIC populations have been used to support the argument for promoting such diets^30^. On the other hand, there is evidence that even in HICs, the protein intake of older people is lower than that of the general population^31^.

Furthermore, despite the indispensable role of high-quality proteins in preserving muscle mass, comprehensive data on the consumption of protein-rich foods and their associations with dietary intake and body composition of community-dwelling older adults are still lacking in SSA^30,32^. In SSA, investigations of ASF consumption among older adults have been limited to the role played by these foods in diet quality and diversity^32^. In these studies, ASFs, together with other food groups, are used to calculate scores for diet quality or diversity by simply counting food groups^32,33^. Using these proxy measures of dietary intake provides little insight into the actual nutrient intake of older people^34^, thus contributing to the limited availability of data on foods and nutrients, such as protein, relevant for healthy aging in SSA. This study aimed to assess ASF consumption and its associations with protein intake and muscle mass in community-dwelling older adults in Gasabo district, City of Kigali, Rwanda.

## Methods

### Study design and setting

This cross-sectional study was conducted as part of a larger project seeking to investigate the role of protein-rich foods and healthy aging in Sub-Saharan Africa. The study was performed in Gasabo district, City of Kigali, Rwanda. The city of Kigali is the capital of Rwanda, with a total population of over 1.7 million^35^. Gasabo district is the largest district (area: 430.3 km^2^) of the City of Kigali, with a population of over half a million (population density: 1237 people/km^2^)^36^. Based on the Rwandan administrative structure, Gasabo district is divided into 15 sectors, 73 cells, and 485 villages^36^.

### Study population, sample size, and sampling procedures

The study was conducted between November 2021 and January 2022 and recruited community-dwelling older adults, following a population-based sampling scheme. The participants were enrolled in the study when they were: (1) aged ≥ 55 years, (2) had lived in Gasabo district for at least six months preceding the interview, and (3) were able to provide informed consent. Patients and older adults residing in institutional settings, such as elderly homes, were not eligible for the study.

The study participants were recruited from Gasabo district. Probabilistic sampling requires listing all units (e.g., people or households) in the study area and randomly selecting the desired units from that list. This approach is preferred for small geographical areas but was financially demanding and unfeasible for this study as the population or households were scattered over a large geographical area. Our study covered all 15 sectors of Gasabo district, which are spread over an area of 400 km^2^. There was no readily available list of older adults or households with older adults from Gasabo district. Thus, a multi-stage cluster random sampling without replacement was used to complete the sampling process. This method helps to develop a sampling frame as the sampling progresses from one stage to another. The 15 sectors of Gasabo district constituted the sampling frame of which 31 villages were randomly selected. In the second stage, households were selected randomly from each village using the list of households (with older adults) developed by the research team in collaboration with community health workers^37^. In the third stage, the participants (older adults aged ≥ 55 years) were randomly selected from eligible households. Since each household was represented by one participant in the study, only one older adult was selected from each of the eligible households. The selection of villages and participants was completed using a table of random numbers generated in Microsoft Excel 2010.

The sample size was calculated priori using G*power software, version 3.0.10^38^, based on the estimated difference (9 g/d, SD: 36) in protein intake between the older adults consuming plant-based diets and those consuming diets rich in animal-sourced foods. A conservative estimate was chosen for this study compared with the difference of about 15 g/d reported by the meta-analysis of intervention studies^39^. Considering alpha = 0.05 and power = 80%, the calculated sample size was 376 older adults. The final targeted sample size was 414 participants, considering a 10% non-response rate.

### Data collection

Trained nutritionists performed the data collection. The collected data included dietary intake, anthropometry, demographic characteristics, food insecurity, and multi-morbidity. The recorded demographic variables were age, sex, marital status, family size and composition, education level, employment status, religion, alcohol consumption, and wealth index. The wealth index was assessed by asking participants to report the respective wealth categories of their households. In Rwanda, all families (households) are classified into wealth categories known as Ubudehe^40^. This classification system is based on a combination of household income, properties, and assets^40^. This classification has four categories, designated as 1 = poorest, 2 = poor, 3 = rich, and 4 = richest^40^. A history of multimorbidity was assessed by asking the participants: “Are you receiving treatment or taking medication for any of these conditions ?”. This self-reported treatment was assessed for six chronic diseases, including high blood pressure, high blood sugar, chronic lung disease, chronic heart disease, cancer, and arthritis. The number of chronic diseases for the participant who answered “yes” was calculated. Participants who reported three or more chronic diseases were classified as having multimorbidity; those who reported fewer than three chronic diseases were classified as having no multimorbidity. Food insecurity among the study population was assessed using the Food Insecurity Experience Scale (FIES) module developed by Food and Agriculture Organization (FAO)^41^. The FIES questionnaire consists of eight items assessing people’s experiences in accessing adequate food during the past 30 days preceding the interview^41^.

#### Anthropometric measurements and body composition

The participants were measured in light clothing and without shoes. Height was measured using a portable stadiometer (Seca, CE 0123) on a flat surface (floor) and recorded to the nearest 0.1 cm. Body weight was measured using a Seca Scale (SECA 874), placed on a flat surface (floor), and recorded to the nearest 0.1 kg. Triceps skinfold thickness (TSF) was measured on the left arm using a Harpenden skin-fold caliper (Baty International, Burgess Hill, England). Circumferences of the mid-upper arm (MUAC), hip (HC), and waist (WC) were measured using non-stretchable length tapes and recorded to the nearest 0.1 cm. WC was measured by placing a non-stretchable midway between the ribs and iliac crest. MUAC was measured by placing a MUAC tape midway between the acromial and olecranon of the arm with the non-dominant hand. Body weight, height, WC, HC, and MUAC were measured in duplicate, whereas TSF was measured in triplicate. Only the average of these measurements was used in the analysis.

Two measures of body compositions, total body skeletal muscle mass (SMM) and mid-arm muscle area (AMA), were estimated from anthropometric variables. SMM (kg) was estimated from body weight, height, age, sex, and ethnicity using the formula described by Lee et al. (2000)^42^. AMA (cm^2^) was estimated based on MUAC and TSF using the formula: MUAC − (3.142 × TSF × 0.1), and sex-specific values were applied^43^.

#### Dietary assessment

Data on food and beverages were collected using the repeated 24-hour recall method during face-to-face interviews. During dietary recalls, participants were first asked to freely list all foods and beverages consumed (from waking up) on the day preceding the interview until they woke up on the day of the interview. In subsequent steps, information on mealtimes, food ingredients, preparation methods, brand names of the foods, amount of the food consumed, and leftovers were recorded. Participants were asked to demonstrate the foods and beverages consumed using household measurements (plates, cups, and spoons). For solid foods, the portion size was estimated using a kitchen scale (Clas Ohlson Kitchen Scale, Model: CFC2028), whereas liquid foods were estimated using graduated cups. If the participants could not easily estimate the portion size of consumed foods, a Kenyan food atlas^44^ was also used to aid their memory. The Kenyan food atlas was chosen based on its close similarity to the foods consumed in Rwanda and the lack of a Rwanda-based tool. Two 24-hour recalls were conducted for each participant on randomly selected non-consecutive days. The recalls included weekdays and weekend days. All interviews were conducted in the local language (Kinyarwanda), and intervals of at least 15 days separated the two recalls to limit the influence of memory on food recalls.

#### Estimation of nutrient intake

No food composition table (FCT) was available for Rwanda during data collection and at the time of reporting this study. An FCT was developed for this study based on the Kenyan Food Composition Table^45^ to calculate nutrient intake. For the foods not covered by the Kenyan food database, food databases from other countries were used, including the Food Composition Table for Western Africa^46^, the Tanzania Food Composition Table^47^, the South African Food Composition Table^48^, and the Food and Nutrient Database for Dietary Studies from the United States Department of Agriculture^49^, in descending order of priority. The priority order for selecting these databases was based on completeness and close similarity to the foods and dishes consumed in Rwanda. Nutrient compositions were also obtained from the food labels and published literature for the foods not covered by these food databases. Foods were converted into energy and nutrients using Compl-EAT software (version 1.0) from Wageningen University and Research, The Netherlands. The average of the two-day dietary recalls was used to calculate macronutrients (protein, fat, and carbohydrates) and energy intake.

### Statistical analyses

For analytical purposes, two dietary groups were created based on ASF consumption status. Participants who reported the consumption of any ASFs in both recalls (meat, dairy, fish, or eggs) were classified as being in the ‘ASF dietary group’, while those who did not report any ASF consumption were classified as being in the ‘plant-based diet’ group. The dietary groups were then coded as “1” for participants who consumed ASFs and “0” for participants who consumed plant-based diets. Sixty participants, corresponding to 15% of the total participants (*n* = 394), were excluded from the study due to extreme (implausible) values of energy intake (< 600 kcal or > 4200 kcal/day).

The background characteristics, anthropometry, and dietary intake data of the participants were described by dietary groups (ASF vs. plant-based diet). A descriptive analysis was also performed to evaluate whether the characteristics of the excluded participants differed from those included in the study.

The association between the independent variables (dietary groups) and outcomes (protein intake and muscle mass) was assessed using inverse propensity score weighting (IPTW) for complex surveys. The propensity score method aims at reducing selection bias by creating a pseudo-population with balanced covariates (confounding) between treatment and control groups^50,51^. The IPTW was performed in three steps. In the first step, the propensity score (PS) was computed using a logit model. The list of confounding variables for the PS model was selected with the help of causal directed acyclic graph (DAG)^52^, considering both measured and unmeasured (appetite/anorexia of aging, depression, and dentition) covariates (Supplementary Figs. 1 and 2). Interaction and quadratic terms were also included in the PS model in order to obtain a more accurate prediction of the probability of being assigned to one of the dietary groups (treatment). To estimate a causal effect, the two dietary groups (ASF and plant-based diet) needed to be comparable (exchangeable) with regard to the confounders^51^. Thus, in the second step, propensity score weights (PSW) were computed for both the ASF and plant-based diet groups. The ASF group was weighted by 1/PS, whereas the plant-based diet group was weighted by 1/(1-PS). Density plots were used to check the distribution of PS and weighted PS separately for the ASF and plant-based dietary groups (Supplementary Figs. 3 and 4). Since some of the observations may gain unreasonably large weight when using IPTW, which can lead to artificially large sample sizes and narrow confidence intervals^51^, weight stabilization was also carried out using the predicted probability of the study population. Thirdly, *teffects* command was used to generate the average treatment effect (ATE) for the study population. The coefficients for ATE and 95% confidence intervals (CIs) were obtained from the IPTW models. The ATE represents the average difference in the outcomes (protein intake and muscle mass) if every participant in the study had consumed ASFs compared to a plant-based diet^51^.

### Sensitivity analysis

Multinomial extension of the inverse propensity scores weighting was used to test the sensitivity of the findings on the associations between ASF consumption and protein intake and muscle mass^53^. To perform this analysis, the ‘ASF dietary group’ was re-categorized into two categories, including participants who consumed only ‘one type of ASFs’ and those who reported ‘two or more ASFs’. These new dietary categories were used to create a new independent (dietary groups) variable, which was coded as “0” for the participants who consumed only plant-based diets, “1” for those who consumed only one type of ASFs, and “2” for participants who consumed two or more ASFs. Sensitivity analyses were also conducted to evaluate whether excluding 15% (n = 60) of the participants who had extreme values of energy intake had an impact on the results or not. All analyses were performed using STATA Software (version 18.0).

Further sensitivity analyses were also conducted to evaluate the extent to which study results could still be affected by unknown confounding using E-values methodology^54^. The E-values are defined as the minimum strength of association that any unmeasured confounders would need to have with both the exposure (dietary groups) and outcomes (protein intake and muscles mass) to be able to attenuate observed association to the null, given the measured covariates^54^. The E-values and 95% CIs were computed using an online calculator^55^.

### Ethical approvals

The study was conducted in accordance with Declaration of Helsinki principles for the research involving human subjects, and it was reviewed and approved by the Institutional Review Board (IRB) of the College of Medicine and Health Sciences, University of Rwanda (Ref. No: 291/CMHS IRB/2021) and the Regional Committee for Medical Research Ethics Western Norway (Ref. No: 163823). Permission to conduct the study was also obtained from Gasabo District’s authorities (Ref. No: 1999/070102/2021). The participants were explained the purpose of the study, and they all provided written informed consent.

## Results

**Fig. 1:**
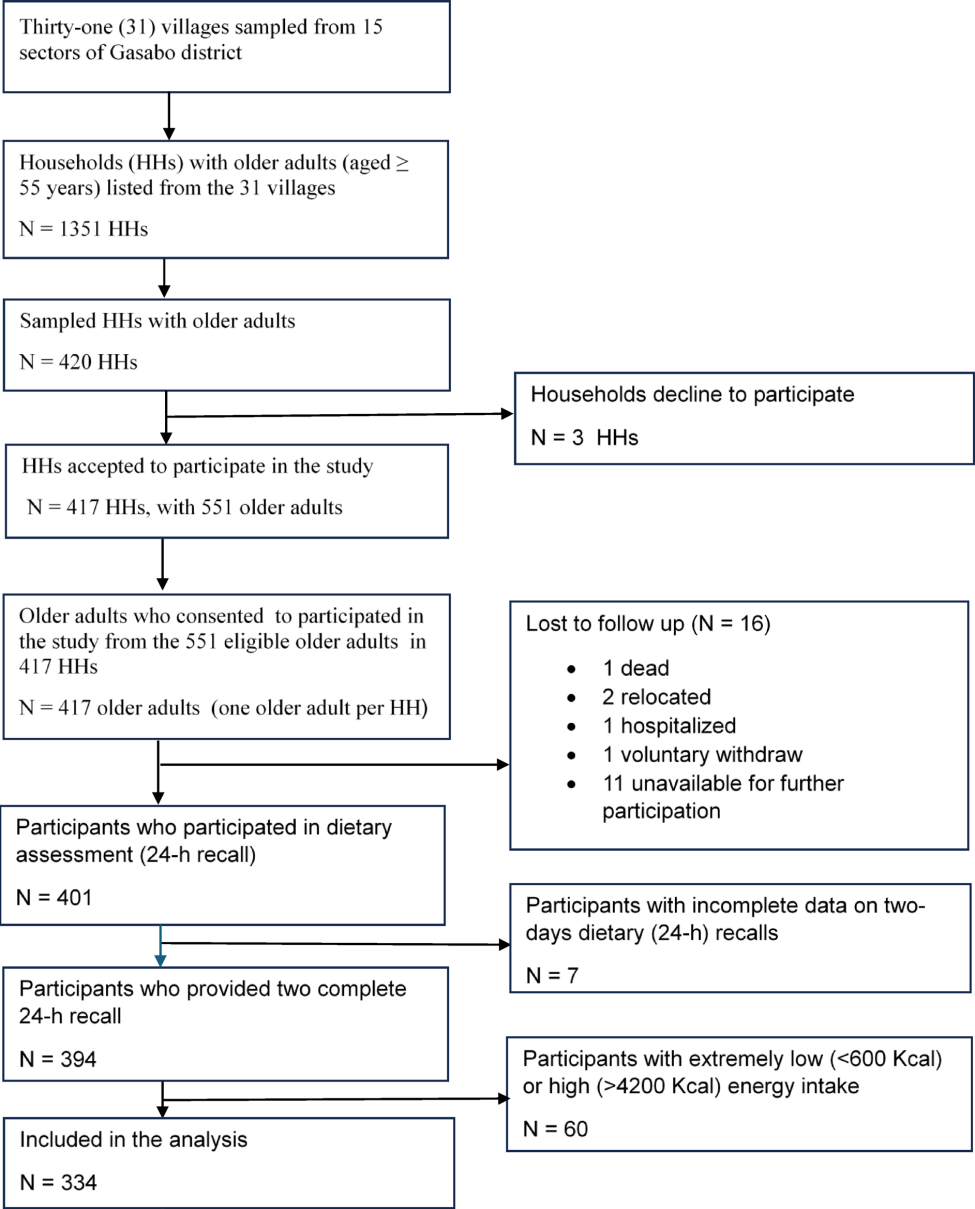
Flowchart for the selection of the participants.

From the initial 417 participants, 334 older adults (254 women and 80 men) were included in the final data set of the present study (Fig. 1). The ages of the participants ranged from 55 to 93 years (median: 65 years), but most of the participants (67%) were aged between 55 and 69 years (Table 1). Only 65.5% (*n* = 222) of the participants reported having attended school, and secondary or higher education was more common in the ASF (35.4%, *n* = 67) than in the plant-based dietary group (10%, *n* = 16). The older adults in the plant-based dietary group were more involved in physical work (86%, *n* = 39) than those in the ASF dietary group (63%, *n* = 24). Food insecurity was also higher in the plant-based (71%) than in the ASF dietary group (33%). The background characteristics of the participants included in the present study were not different from those who were excluded (Supplementary Table 1).

**Table 1:** Background characteristics of the study population stratified by dietary groups in median (IQR: 25%, 75%) or count (%).

	Plant-based dietary group (*N* = 145)	ASF dietary group (*N* = 189)	*P*-value
Age, median (IQR)	66 (60–72)	65 (59–72)	0.285
Age groups, n (%)			
55–69 years	96 (66.2)	129 (68.3)	0.860
70 + years	49 (33.8)	60 (31.7)	
Sex, n (%)			
Male	36 (24.8)	44 (23.3)	0.652
Female	109 (75.2)	145 (76.7)	
Marital status, n (%)			
Single/separated/divorced	57 (39.3)	73 (38.6)	0.890
Cohabiting/married	88 (60.7)	116 (61.4)	
Family size, median (IQR)	4 (2–6)	4 (3–6)	0.343
Categories of family size, n (%)			
Small family	90 (62.1)	113 (59.8)	0.763
Large family	55 (37.9)	76 (40.2)	
School attendance, n (%)			
Did not attend school	68 (46.9)	44 (23.3)	< 0.001
Primary education	61 (42.1)	78 (41.3)	
Secondary or higher education	16 (11.0)	67 (35.4)	
Religion, n (%)			
Christian	142 (97.9)	182 (96.3)	0.509
Others	3 (2.1)	7 (3.7)	
Working/employment status, n (%)			
No	99 (68.3)	139 (73.5)	0.001
Yes	46 (31.7)	50 (26.5)	
Occupation involving physical work, (%)			
No physical work/job	6 (13.3)	14 (36.8)	0.124
Physical work/job	39 (86.7)	24 (63.2)	
Wealth categories, n (%)			
High income	51 (35.2)	97 (51.3)	< 0.001
Low income	94 (64.8)	92 (48.7)	
Family conditions, n (%)			
Live with other people in the HH	123 (84.8)	169 (89.4)	0.147
Alone in the household	22 (15.2)	20 (10.6)	
Presence of other older adults in the HH, n (%)			
No	101 (69.7)	136 (72.0)	0.935
Living with other older adults	44 (30.3)	53 (28.0)	
Children under 5 years old in the HH, n (%)			
No	116 (80.0)	154 (81.5)	0.611
One or more	29 (20.0)	35 (18.5)	
NCDs, n (%)			
Less than three NCDS	142 (97.9%)	178 (94.2)	0.047
Three or more NCDS	3 (2.1)	11 (5.8)	
Food security status, n (%)			
Food secure	42 (29.0)	126 (66.7)	< 0.001
Moderate food insecurity	34 (23.4)	24 (12.7)	
Severe food insecurity	69 (47.6)	39 (20.6)	
Drink alcohol, n (%)			
No	78 (53.8)	119 (63.0)	0.675
Yes	67 (46.2)	70 (37.0)	

*HH* household, *NCD* non-communicable disease IQR (25%, 75%), Interquartile range.

### Dietary (nutrients and energy) intake

The data on energy and macronutrient (protein, carbohydrate, and fat) intake, stratified by dietary groups, are presented in Table 2. Only slightly more than half (56%, *n* = 189) of the older adults consumed ASFs. Meat and dairy (milk) were the main sources of animal proteins, each contributing to over 5 g of protein daily. Pulse and legumes were the main sources of plant-based proteins, contributing to over 6 g of daily protein intake. Overall, protein (38 g/d, SD = 18) and energy intake (1,314, SD = 488) were higher in the ASFs dietary group than in the plant-based dietary group (protein intake: 24 g/d, SD = 9 and energy: 1,107 kcal/d, SD = 433). The older adults who consumed ASFs also reported higher fat intake (45 g/d, SD = 27) than those who consumed plant-based diets (28 g/d, SD = 22). On the contrary, there was no difference in carbohydrate intake between older adults who consumed ASFs (173 g/d, SD = 62) and those who consumed plant-based diets (169 g/d, SD = 84).

**Table 2:** Energy (kcal/d) and macronutrient intake (g/d) of men and women stratified by dietary groups in mean (SD).

Dietary intake	Plant-based dietary group (*N* = 189)	ASF dietary group (*N* = 145)	
Energy intake (kcal/d)	1107 (433)	1314 (488)	< 0.001
Carbohydrates (g/d)	169 (84)	173 (62)	0.021
Fat (g/d)	28 (22)	45 (27)	< 0.001
Protein intake (g/d)	24 (9)	38 (18)	< 0.001
Animal protein (g/d)			
Meat and poultry	0 (0)	5 (11)	< 0.001
Fish and sea foods	0 (0)	3 (6)	< 0.001
Milk and dairy	0 (0)	5 (7)	< 0.001
Eggs	0 (0)	0 (1)	0.010
Plant protein (g/d)			
Pulse and legumes	9 (5)	6 (4)	0.006
Grain and cereals	5 (5)	6 (5)	< 0.001
Roots and tubers	6 (4)	5 (3)	0.082
Vegetables	5 (4)	6 (4)	0.005
Fruits	0 (1)	0 (0)	0.012
Other protein sources	0 (1)	0 (0)	0.673

Others (alcoholic drinks, juice, soft drinks, oil, spices/herbs, and mixed protein-source foods).

*g/d* gram per day; standard deviation.

### Association between ASF consumption and protein intake

**Fig. 2:**
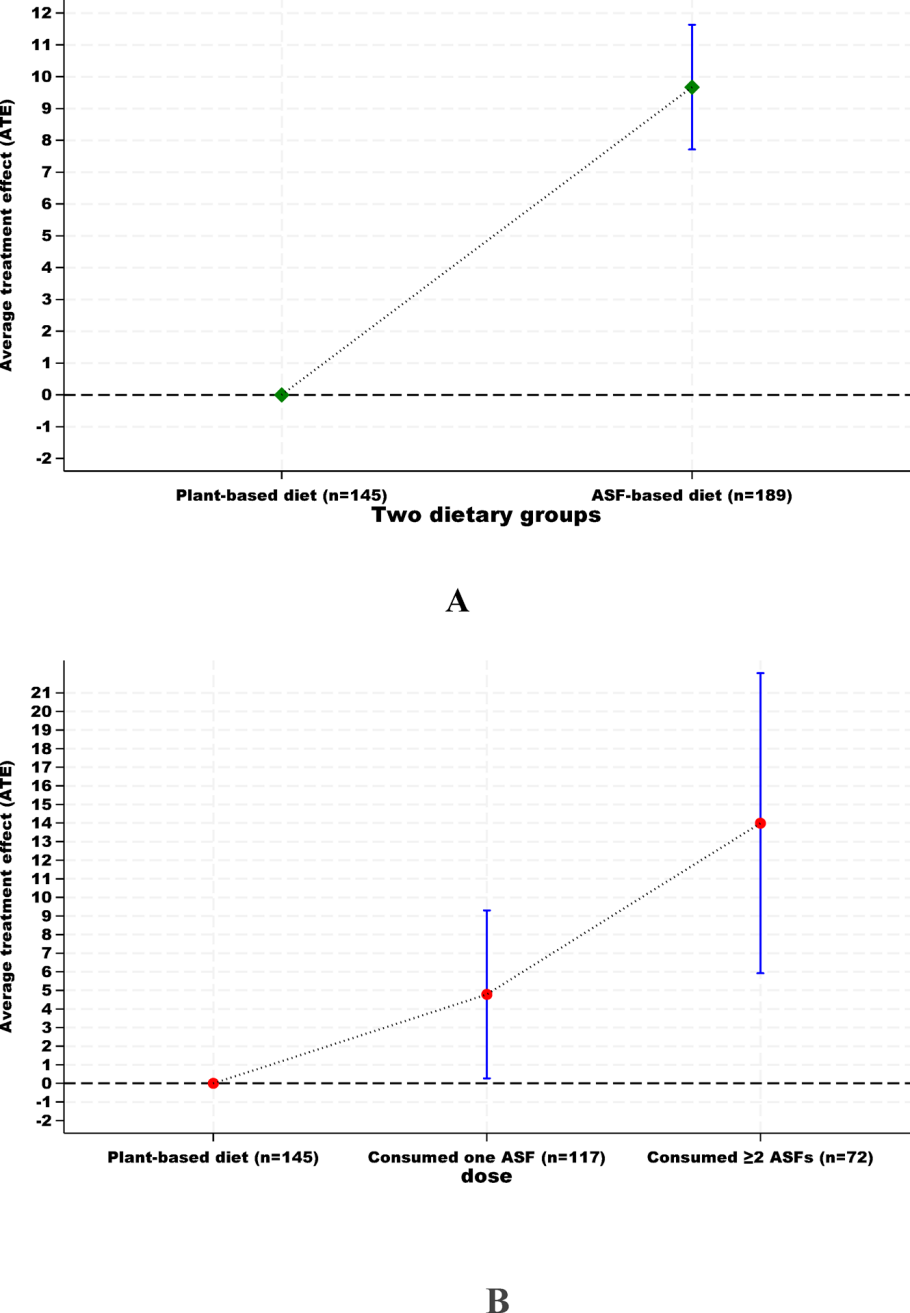
The association between ASF consumption and protein intake in the study population, using two dietary groups (**A**) and three dietary groups (**B**) as an independent (exposure) variable. The plant-based dietary group is used as the reference group. Data are presented as average treatment effect (ATE) in g/d, with 95% confidence intervals (CIs).

Inverse propensity score weighting (IPTW) analyses were performed to investigate the association between ASF consumption and protein intake. The results showed that ASF consumption was associated with a 9.6 g/d (95% CI, 6.8 to 12.4) increase in protein intake in the study population (Fig. 2A). The analysis also showed that adding one ASF and two or more ASFs to the diet was proportionally associated with an increase in protein intake in the study population (ATE: 4.7 g/d; 95% CI, 0.2 to 9.3 and ATE: 13.9 g/d; 95% CI, 5.9 to 22.0, respectively), Fig. 2B. Sensitivity analyses showed that including the participants with extreme values of energy did not change the results on the association between ASF consumption and protein intake (Supplementary Fig. 5). The E-value was 36.39 for the estimated difference in protein intake and 26.95 for the lower confidence limit (Supplementary Fig. 6).

### Anthropometric indices and body composition

Anthropometric indices and body composition values are presented in Table 3. The older adults who consumed ASFs were heavier (66 kg, SD = 18 vs. 58 kg, SD = 13) and had higher TSF (20 mm, SD = 16 vs. 16 mm, SD = 9), HC (99 cm, SD = 16 vs. 91 cm, SD = 12), WC (92 cm, SD = 16 vs. 86 cm, SD = 15), MUAC (29 cm, SD = 5 vs. 26 cm, SD = 4), and SMM (48 kg, SD = 10 vs. 44 kg, SD = 7) than those of older adults who consumed plant-based diets.

**Table 3:** Anthropometric and body composition of the study population stratified by dietary groups in mean (SD).

Anthropometry and body composition	Plant-based dietary group (*N* = 189)	ASF dietary group (*N* = 145)	
Height^1^	159 (9)	161 (9)	0.005
Weight^2^	58 (13)	66 (18)	< 0.001
TSF^3^	16 (9)	20 (12)	< 0.001
HC^1^	91 (12)	99 (16)	< 0.001
WC^1^	86 (15)	92 (16)	0.005
MUAC^1^	26 (4)	29 (5)	< 0.001
Skeletal muscle mass (SMM)^2^	44 (7)	48 (10)	< 0.001
Mid-arm muscle area (AMA)^2^	26 (4)	28 (5)	< 0.001

*TSF* triceps skinfold, *HC* hip circumference; waist circumference, *MUAC* mid-upper arm circumference.

^1^Values are presented in kilogram (kg).

^2^Values are presented in millimeters (mm).

^3^Values are presented in centimeter (cm).

### Association between ASF consumption and skeletal muscle mass (SMM)

**Fig. 3:**
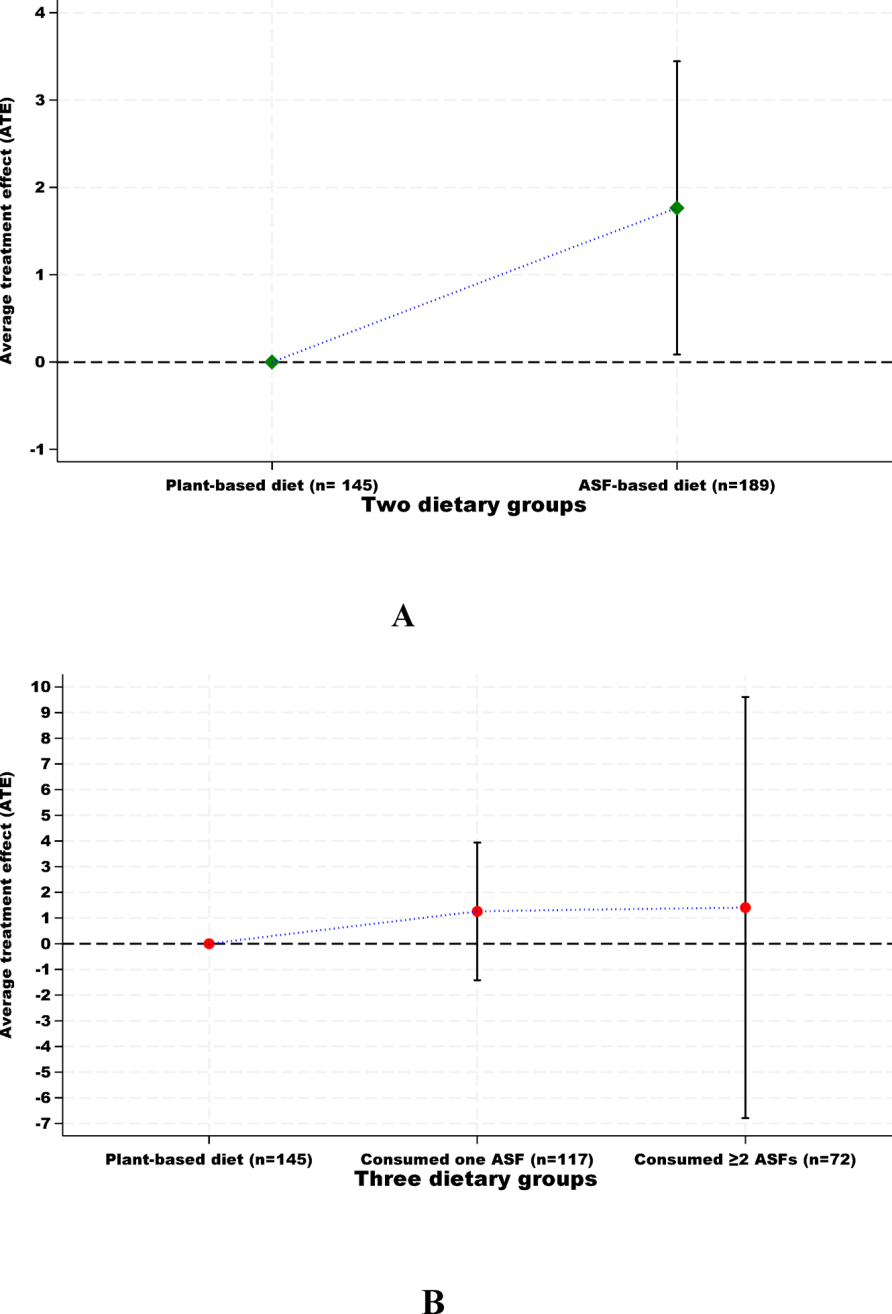
The association between ASF consumption and muscle mass in the study population, using two dietary groups (**A**) and three dietary groups (**B**) as an independent (exposure) variable. The plant-based dietary group was used as the reference group. Data are presented as average treatment effect (ATE) in kg, with 95% confidence intervals (CIs).

Inverse propensity score weighting (IPTW) analyses were performed to investigate the association between ASF consumption and muscle mass. The results show that ASF consumption was associated with an increase in muscle mass of the study population (ATE: 1.7 kg; 95% CI, 0.0 to 3.4), Fig. 3A. The analysis also showed that adding one and two or more ASFs in the diet was not associated with an increase in muscle mass of the study population (ATE: 1.2 kg; 95% CI, − 1.4 to 3.9 and ATE: 1.4 kg; 95% CI, − 0.6 to 9.6, respectively), Fig. 3B. Sensitivity analyses showed that including the participants with extreme values of energy did not change the results on the association between ASF consumption and muscle mass (Supplementary Fig. 7). The E-value was 12.52 for the estimated difference in muscle mass and 9.7 for the lower confidence limit (Supplementary Fig. 8).

## Discussion

We conducted a cross-sectional study to assess the associations between ASF consumption and protein intake and muscle mass in community-dwelling older adults in Gasabo district. The study found that ASF consumption was low among older adults, but that it was positively associated with protein intake and muscle mass.

In our study, only about half of the older adults consumed ASFs. There are no comparable dietary intake data in Rwanda, but the data from other Sub-Saharan African (SSA) countries show that the diets of older adults are very low in ASFs^56^. The consumption of ASFs was shown to be low in the Sodo Zuraya region of Ethiopia, where only 15%, 42%, and 60% of the older adults consumed eggs, meat, and dairy products^57^, respectively. Low consumption of ASFs has also been reported in the Republic of Congo and the Central Africa Republic, where only 55% of older adults in the CAR and 15% in the ROC consumed meat, fish, or eggs^58^. Eggs are considered to be one of the most affordable and least environmentally damaging dietary sources of animal proteins^59^. In our study, however, eggs were the least-consumed ASFs, and future studies are needed to investigate the underlying motives for choosing between animal protein sources in this population.

In the present study, increasing the number of ASFs in the diet of older adults was proportionally associated with an increase in total protein intake and muscle mass. Previous studies, mostly from HICs, have shown that older adults consuming plant-based diets may have low protein intake and perform poorly on different outcomes of muscle mass and function^60,61^. Data from the Canadian National Dietary Survey showed that replacing animal-based with plant-based proteins proportionally reduced the total protein intake among older adults (aged > 70 years)^62^. A cross-sectional study of elderly Chinese vegetarians and omnivores who were matched on age and sex found that vegetarians had a lower energy, fat, and protein intake^63^.

Intervention and observational studies, mostly from HICs, have also shown that ASF consumption is positively associated with lean body mass^64^. In a 12-week randomized trial, older adults who consumed eggs had higher lean body mass than those who consumed diets without eggs^65^. A meta-analysis of the effects of plant protein vs. animal protein on lean mass reported that dietary sources of protein did not affect the total lean mass among adults (> 50 years)^66^. This meta-analysis, however, reported a favorable effect of animal protein on lean mass in young adults (< 50 years)^66^. In a cross-sectional study from Milan, older adults who were classified into the highest tertiles of animal protein intake had higher muscle mass and function compared with older adults into the lowest tertiles^67^. Consumption of ASFs reduced the risk of low muscle mass by 60% compared with consumption of plant-based diets in a cohort of Chinese older adults (aged ≥ 65 years) after four years of follow-up^68^.

This study has strengths and limitations. The cross-sectional nature of the study limited our ability to account for time-varying confounding. Similarly, cross-sectional data also suffer from the possibility of reverse causation. Therefore, only associations, not causation, can be inferred from our findings. The findings of this study cannot be generalized to older adults outside of Gasabo district as the study did not include the participants from other districts. Dietary groups (exposure) were constructed based on self-reported consumption of ASFs. Self-reported data are prone to desirability bias, which may lead to misclassification^69^. However, ASFs were assessed as part of the food and beverages consumed by the participants, and it is unlikely that the participants would have reported the consumption of any ASFs if they had not. ASFs are distinct from other foods, reducing any risk of bias during the assessment and categorization of dietary groups. The data presented in this study were collected in November and December, which is a pre-harvest period (lean season) in Rwanda. The period of data collection was also during the COVID-19 pandemic with intermittent lockdowns. It has been shown that over 20,000 families were severely affected by COVID-19 and were depending on in-kind food support from the government in 2020^70,71^. These factors might have contributed to the low variety and quantity of consumed foods^72^. Thus, post-COVID-19 studies are needed to capture habitual dietary patterns and seasonal variations to provide a full picture of nutrition in older adults in Rwanda. There was no national food composition database available from Rwanda at the time this study was conducted. We had to use food databases from neighboring countries. It has been shown that nutrient content in FCTs may differ between countries and regions due to various factors, including methodological variations in the production of FCTs and differences in climatic and topographic conditions^73,74^. Thus, using foreign FCTs may lead to either under- or over-estimation of nutrient intake.

To our knowledge, this is the first study to assess the association between ASF consumption and both protein intake and body composition in community-dwelling older adults in the East African region. The anthropometric and dietary assessments were conducted by trained nutritionists. The use of IPTW for complex surveys also reduced the risk of selection bias. Exploration of the residual confounding suggested that relatively substantial unmeasured confounding would be needed to fully explain away the observed associations between ASFs consumption and both protein intake and muscle mass, thereby strengthening the study’s internal validity.

### Public health and research implications

Despite the limitations, the findings of this study provide an important contribution with relevant implications for both research and policy on healthy aging. Firstly, this study showed that there are gaps in the dietary intake of older adults, highlighting the need for monitoring the diet and nutrition status of the older population within and beyond urban settings of Rwanda. Secondly, the findings also showed that the inclusion of ASFs in the diet of community-dwelling older adults might be beneficial not only for the preservation of muscle mass but also for achieving energy (calorie) and nutrient intake. Since energy and food consumption are generally low in the older population, optimization studies are also needed to identify the options of food combinations that can be used to optimize protein intake of older adults, while considering affordability, acceptability, and agro-ecological constraints. Thirdly, future studies are also needed to identify factors influencing ASF consumption and overall dietary intake among older adults in Rwanda. Having such data will help to identify specific strategies that can be used to improve dietary intake and overall nutrition of older population in Rwanda.

## Conclusion

The consumption of ASFs was generally low among community-dwelling older adults. However, older adults who consumed ASFs had improved protein intake and muscle mass compared with those who consumed plant-based diets. Large and prospective studies, extending beyond urban settings, are needed to identify factors that can be targeted to improve ASF consumption and overall dietary intake among older adults in Rwanda.

## Electronic supplementary material

Below is the link to the electronic supplementary material.

Supplementary Material 1

## Data Availability

The dataset and codes used for this study are available from the corresponding author up on reasonable request.
