# Supplementary material for: Protein intake and muscle mass of community-dwelling older adults: a cross-sectional study in Kigali, Rwanda
Source: Sci Rep. 2025 May 24;15:18097. doi: 10.1038/s41598-025-03291-2 (PMC12103568; doi:10.1038/s41598-025-03291-2)
Supplement: Supplementary file 1 — Supplementary Material 1 [file 41598_2025_3291_MOESM1_ESM.pdf]

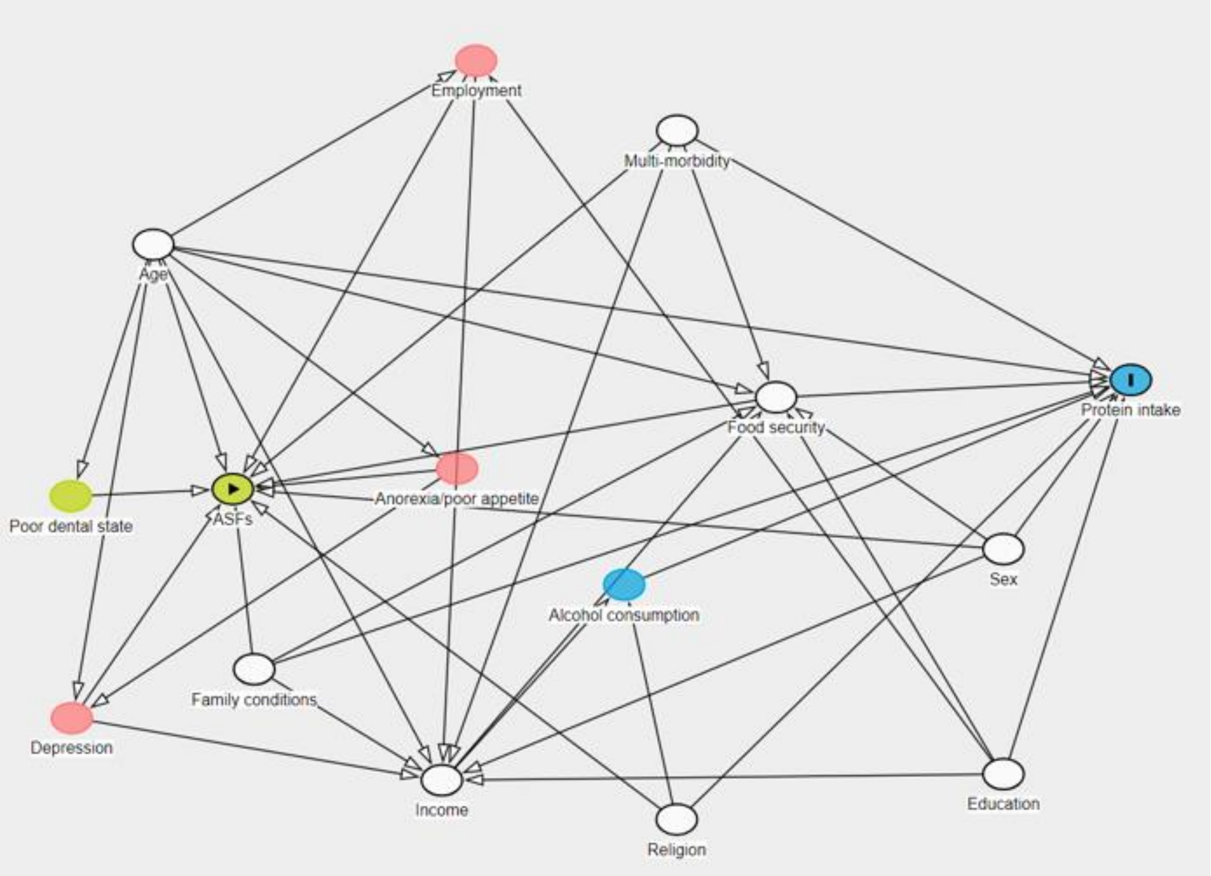

Supplementary Figure 1: Directed acyclic graph depicting an assumption on the causal structure of dependence between the exposures (ASF consumption), outcome (protein intake), and covariates.

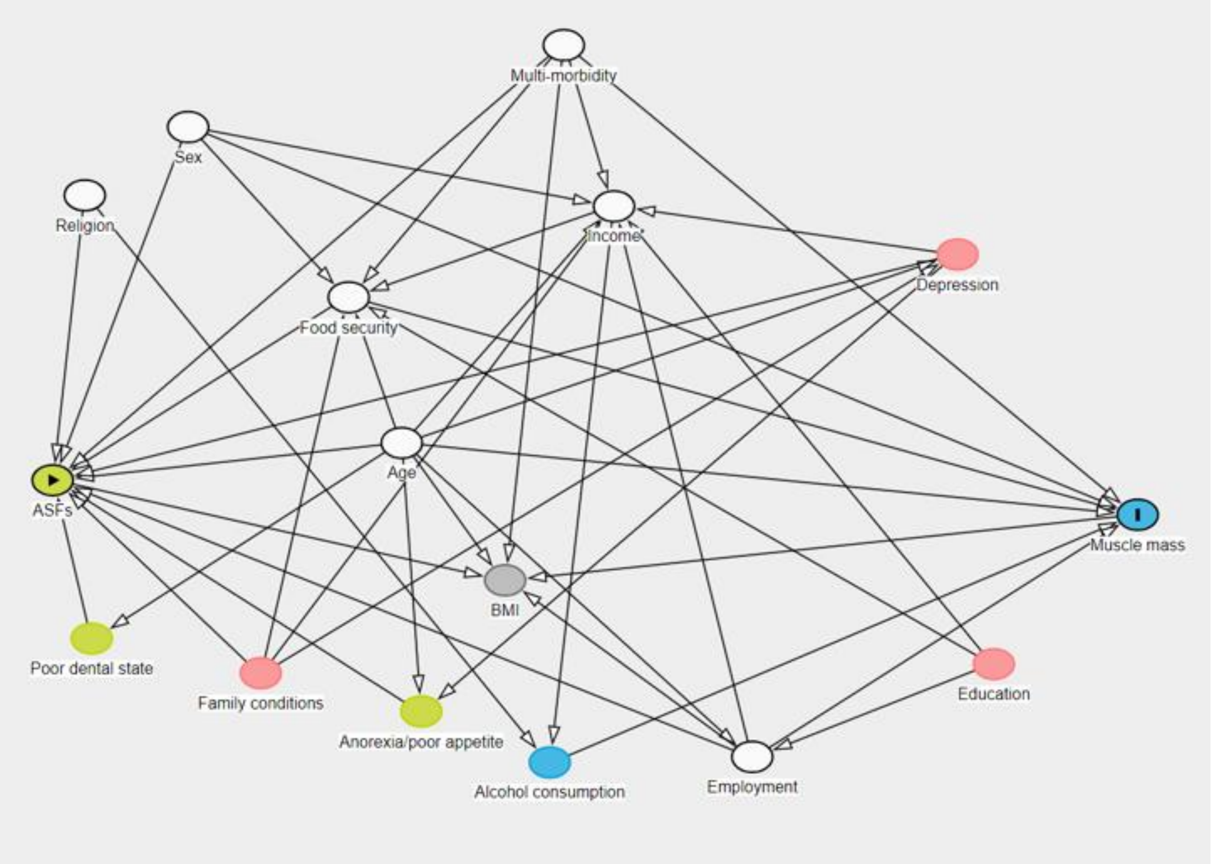

Supplementary Figure 2: Directed acyclic graph depicting an assumption on the causal structure of dependence between the exposure (ASF consumption), outcome (muscle mass), and covariates.

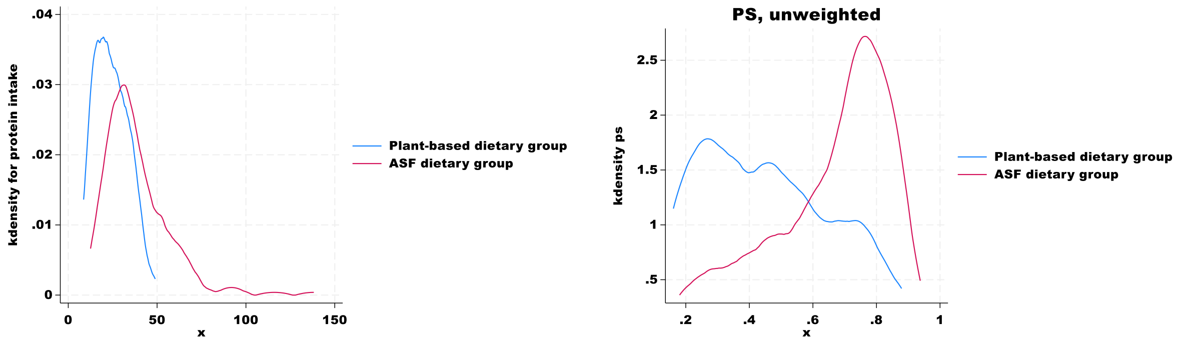

A **B**

Protein intake and muscle mass of community-dwelling older adults: A cross-sectional study in Kigali, Rwanda. **Online supplementary materials**

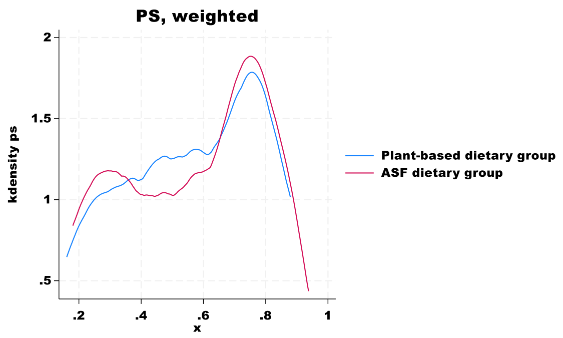

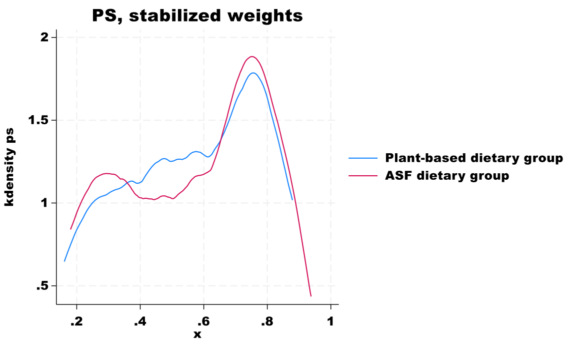

C

D

Supplementary Figure 3: A) Distribution of protein intake, **B**) Distribution propensity score (PS, unweighted), **C**) Distribution of weighted PS, **D)** Distribution of PS with stabilized weight.

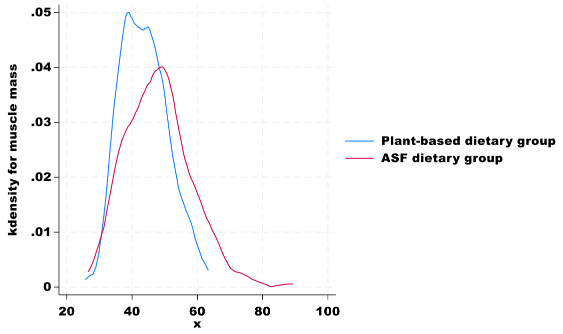

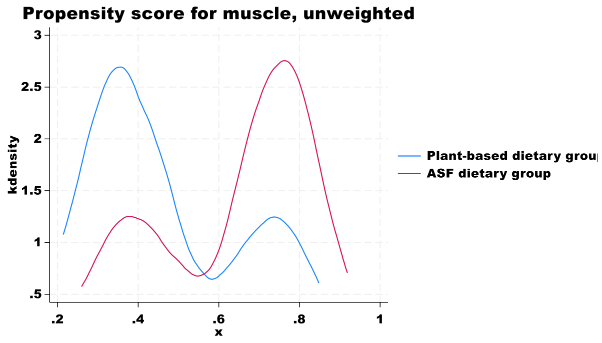

A

B

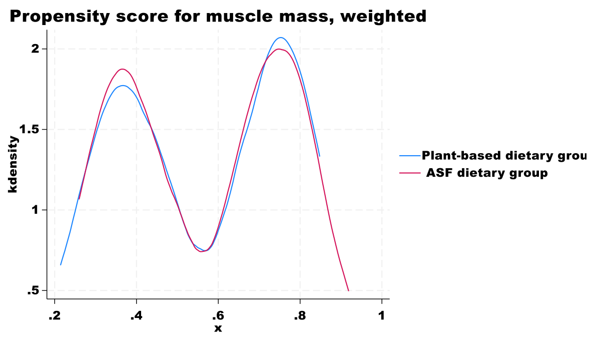

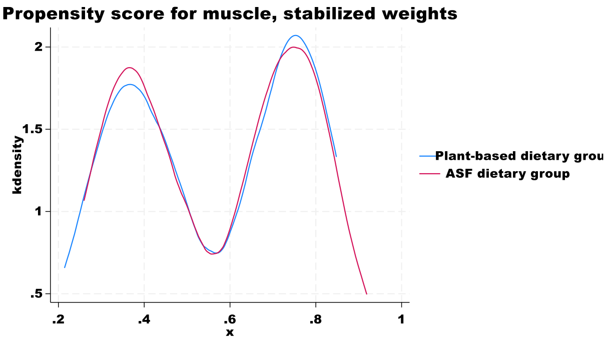

C

D

Supplementary Figure 4: **A**) Distribution of muscle mass, **B**) Distribution of propensity score (PS, unweighted), **C**) Distribution of weighted PS, **D)** Distribution of PS with stabilized weight.

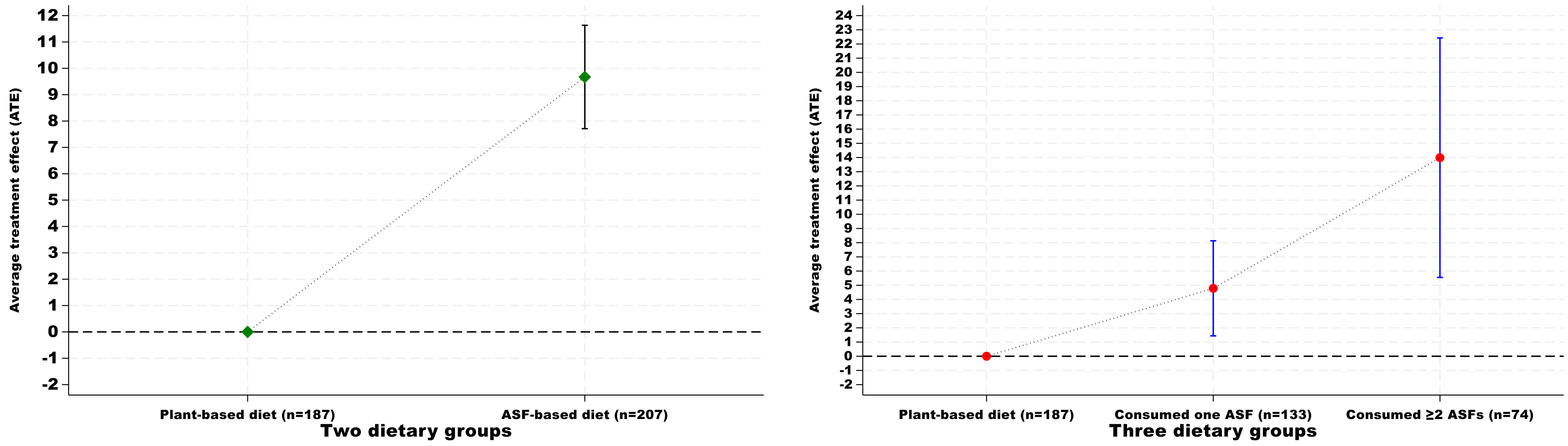

A

B

Supplementary Figure 5. The association between ASF consumption and protein intake in the study population, using two dietary groups (A) and three dietary groups (B) as independent (exposure) variable. Data are presented as average treatment effect (ATE) in g/d, with 95% confidence intervals (CIs). The analysis included the participants with extreme energy values.

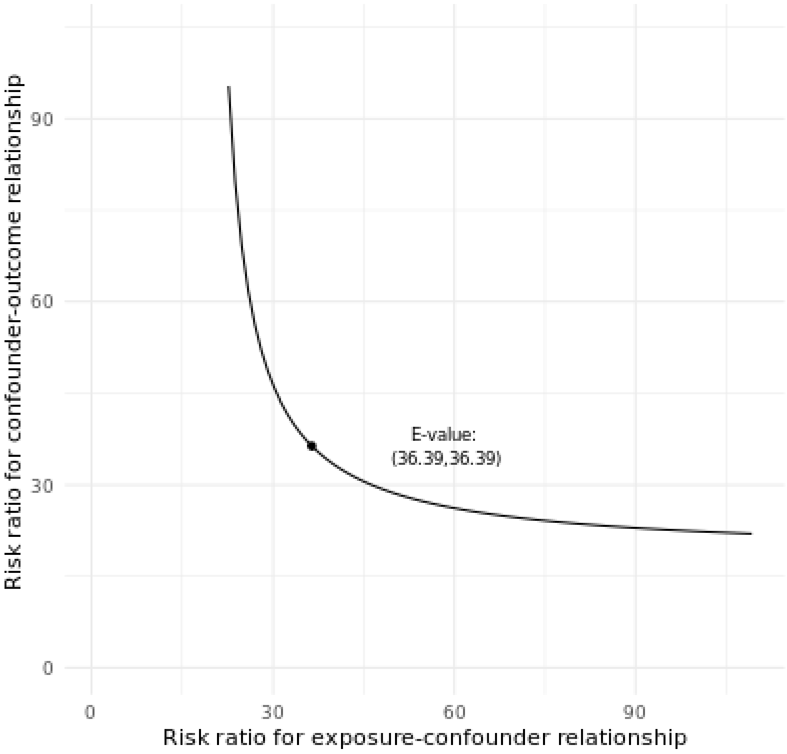

Supplementary Figure 6. E-value and lower confidence limit for the association between ASF consumption and protein intake**.** Each point along the curve defines a joint relationship between the two sensitivity parameters that could potentially explain away the association between ASF consumption and protein intake.

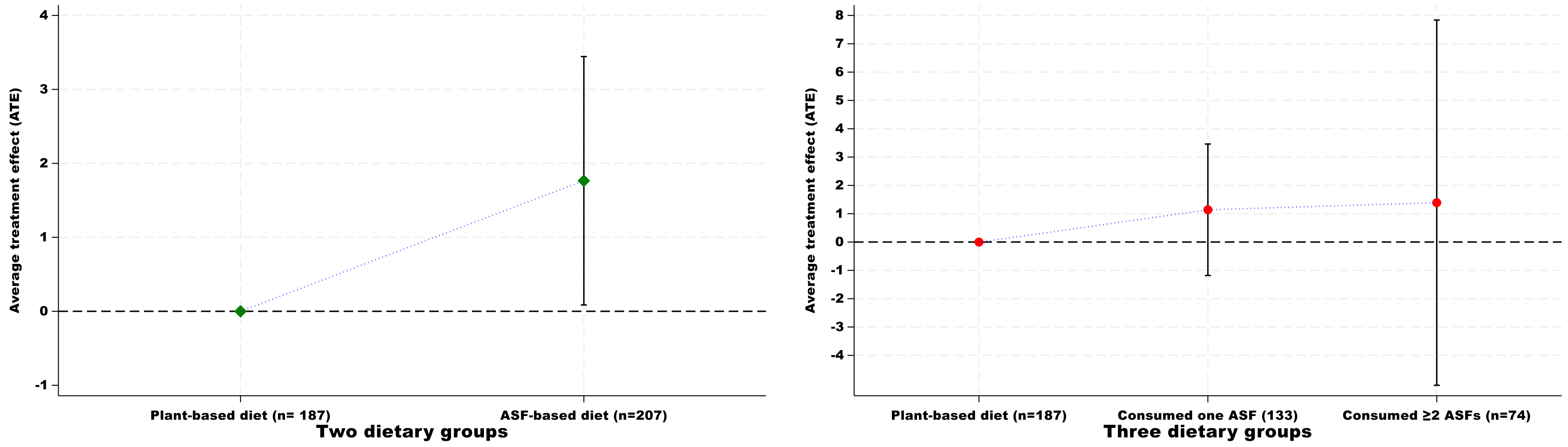

A

B

Supplementary Figure 7. The association between ASF consumption and muscle mass in the study population, using two dietary groups (**A**) and three dietary groups (**B**) as an independent (exposure) variable. Data are presented as average treatment effect (ATE) in kg, with 95% confidence intervals (CIs). The analysis included the participants with extreme energy values.

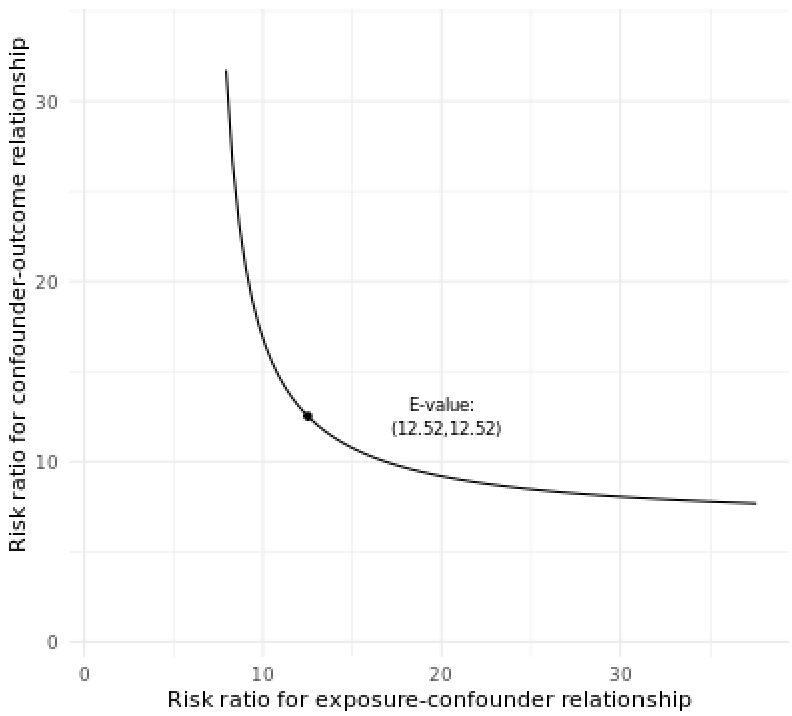

Supplementary Figure 8. E-values and lower confidence limit for the association between ASF consumption and muscle mass. Each point along the curve defines a joint relationship between the two sensitivity parameters that could potentially explain away the association between ASF consumption and muscle mass.

Supplementary Table 1. Background characteristics of the participants excluded from the study in median (IQR: 25%, 75%) or count (%)

	**All (N = 60)**	**Plant-based dietary group (N = 42)**	**ASF dietary group (n = 18)**
Age, median (IQR)	67 (62-73)	66 (60-72)	69 (65-77)
Age groups, n(%)			
55 - 69 years	38 (63.3)	28 (66.7)	10 (55.6)
70+ years	22 (36.7)	14 (33.3)	8 (44.4)
Sex, n(%)			
Male	15 (25.0)	11 (26.2)	4 (22.2)
Female	45 (75.0)	31 (73.8)	14 (77.8)
Marital status, n (%)			
single /Separated/divorced	21 (35.0)	14 (33.3)	7 (38.9)
Cohabiting/ Married	39 (65.0)	28 (66.7)	11 (61.1)
Family size, median (IQR)	4 (3-6)	4 (2-6)	4 (3-6)
Categories of family size, n(%)			
Small family	32 (53.3)	23 (54.8)	9 (50.0)
Large family	28 (46.7)	19 (45.2)	9 (50.0)
Education, n (%)			
No education	37 (61.7)	28 (66.7)	9 (50.0)
Primary education	18 (30.0)	10 (23.8)	8 (44.4)
Secondary or higher	5 (8.3)	4 (9.5)	1 (5.6)
Religion, n(%)			
Christian	57 (95.0)	40 (95.2)	17 (94.4)
Others	3 (5.0)	2 (4.8)	1 (5.6)
Employment status, n(%)			
Unemployed	38 (63.3)	25 (59.5)	13 (72.2)
Work in non-physical labor/job	4 (6.7)	3 (7.1)	1 (5.6)
Work in physical labor/job	18 (30.0)	14 (33.3)	4 (22.2)
Family conditions, n (%)			
Live with other people in the Household	53 (88.3)	36 (85.7)	17 (94.4)
Alone in the household	7 (11.7)	6 (14.3)	1 (5.6)
Presence of other older adults in the HH, n(%)			
Without any older adults	44 (73.3)	32 (76.2)	12 (66.7)
Living with other older adults	16 (26.7)	10 (23.8)	6 (33.3)
Children under 5 years old in the HH, n(%)			
No under5yrs children	44 (73.3)	31 (73.8)	13 (72.2)
One or more under5yrs children	16 (26.7)	11 (26.2)	5 (27.8)
Multiple NCDs, n(%)			
Less than three NCDS	60 (100.0)	42 (100.0)	18 (100.0)
Food insecurity, n(%)			
Food secure	8 (13.3)	6 (14.3)	2 (11.1)
Moderate food insecurity	14 (23.3)	9 (21.4)	5 (27.8)
Severe food insecurity	38 (63.3)	27 (64.3)	11 (61.1)
Drink alcohol, n(%)			
No	60 (100.0)	42 (100.0)	18 (100.0)

HH, household; IQR (25%, 75%), Interquartile range
